# Short-Term Outcomes in Neonates Born to Diabetic Mothers During the Coronavirus Disease 2019 Pandemic: A Descriptive Study

**DOI:** 10.1055/a-2860-5934

**Published:** 2026-06-16

**Authors:** Anfal Awwadh Alshalawi, Michael R. Miller, Malack Turki Alotayan, Khalid Faisal Alkahtani, Adnan Hadid, Nada Alayed, Lana A. Shaiba, Orlando Pereira Da Silva

**Affiliations:** 1 Department of Perinatology-NeonatologyWestern UniversityLondaonOntarioCanada; 2 Children’s Health Research InstituteLondonOntarioCanada; 3 Department of PediatricsKing Saud University Medical CityRiyadhKSA; 4 Department of PediatricsCollege of Medicine, King Saud UniversityRiyadhKSA; 5 Department of Obstetrics and GynecologyCollege of Medicine, King Saud UniversityRiyadhKSA; 6 Department of Pediatrics, Epidemiology and BiostatisticsUniversity of Western OntarioLONDONOntarioCanada

**Keywords:** neonates, diabetic, mothers, COVID, pandemic

## Abstract

**Background** Gestational diabetes mellitus (GDM) negatively affects newborns. Several studies have reported new-onset diabetes associated with coronavirus disease 2019 (COVID-19). Multiple pathophysiological mechanisms have been proposed to explain the relationship between COVID-19 and glycemic dysregulation in neonates. We assessed neonatal outcomes among mothers with diabetes during the COVID-19 pandemic.

**Methods** This retrospective descriptive study included mothers with diabetes (Type 1, Type 2, and GDM) who were COVID-19 positive at least 24 h before delivery and those who were negative, to compare newborn outcomes. The study was conducted at King Saud University Medical City, Riyadh, from January 2020 to December 2022.

**Results** A total of 669 diabetic mothers were included; 23 were COVID-19 positive at delivery. Sixteen had Type 1, 59 had Type 2, and 572 had GDM. Most managed diabetes by diet. Among newborns, 128 (18.6%) in the COVID-19-negative group and 8 (34.8%) in the COVID-19-positive group developed hypoglycemia. Of these, 105 required neonatal intensive care unit (NICU) admission. Most were treated with intravenous dextrose, and five needed glucagon. Mean NICU stay was 12.9 days for negatives and 7 days for positives. One intrauterine fetal death occurred in the negative group.

**Conclusion** Hypoglycemia occurred mainly within the 1 h and resolved with early feeding. Immediate postnatal feeding and screening at 2 h are recommended. No significant differences were found in short-term neonatal outcomes between COVID-19-positive and negative mothers.

## Introduction

The prevalence of diabetes in pregnancy, including gestational diabetes, is very high in Saudi Arabia. 1 Gestational diabetes is a global complication of pregnancy, and its prevalence has recently increased, as reported by the National Vital Statistics in the United States. A 20% increase in gestational diabetes mellitus (GDM) was observed between 2016 and 2020. 2 In Saudi Arabia, the prevalence of diabetes is much higher than that in other countries, with GDM rates among Saudi women ranging from 24% to 39%. 3 4 5 6

GDM has negative effects on both mothers and infants, such as premature birth, small gestational age, macrosomia, hypoglycemia, and increased risk of malformations. 2 7 One of the major effects on infants is hypoglycemia, which often leads to neonatal intensive care unit (NICU) admission and has long-term consequences. Several studies have demonstrated the long-term effects of neonatal hypoglycemia. Interestingly, mild hypoglycemia in moderately preterm infants has been associated with higher rates of developmental delay. 8 Brain magnetic resonance imaging scans of 35-week term infants with severe hypoglycemia showed white matter changes, and 65% of these infants exhibited neurodevelopmental impairments at 18 months of age. 9 Additionally, a multicenter study conducted by Lucas et al. suggested that a blood glucose concentration of <47 mg/dL was the critical threshold associated with adverse neurodevelopmental outcomes. 10

During the coronavirus disease 2019 (COVID-19) pandemic, several studies have reported new-onset diabetes associated with COVID-19 and post-COVID-19 syndrome, also known as Long COVID. 11 12 Although there are multiple potential causes, such as organ dysfunction and effects of hospitalization and medications, the exact cause remains unknown. Pregnant women with both COVID-19 and GDM were almost three times more likely to be admitted to the intensive care unit than women without GDM. 13 Several complications can develop in newborns, but the most prevalent include NICU admission, fetal distress, intrauterine growth retardation, premature birth, spontaneous abortion, and multisystem inflammatory syndrome in children. 11

Several pathophysiological mechanisms have been postulated to explain the association between COVID-19 infection and neonatal hypoglycemia. First, the severe acute respiratory syndrome coronavirus 2 (SARS-CoV-2) virus can directly damage pancreatic β-cells through angiotensin-converting enzyme 2 (ACE2) receptors, which are abundantly expressed on islet cells, potentially leading to impaired insulin regulation and maternal hyperglycemia with consequent fetal hyperinsulinism. 14 15 Second, the systemic inflammatory response triggered by COVID-19 infection, characterized by elevated proinflammatory cytokines such as interleukin-6 and tumor necrosis factor-α, can induce maternal insulin resistance and stress hyperglycemia, which in turn stimulates excessive fetal insulin production and predisposes neonates to hypoglycemia after delivery when the maternal glucose supply is abruptly discontinued. 16 17 Third, corticosteroid therapy, commonly used in the management of moderate-to-severe COVID-19, can exacerbate maternal hyperglycemia; however, in our cohort, the majority of COVID-19-positive mothers were treated with paracetamol alone, which attenuates the contribution of iatrogenic causes. 18 Finally, the physiological stress of concurrent infection may activate the hypothalamic–pituitary–adrenal (HPA) axis, resulting in cortisol-mediated glycemic excursions that further drive fetal hyperinsulinism and postnatal hypoglycemia. 19

### Objective

This study aimed to investigate the short-term outcomes of newborns born to women with diabetes during the COVID-19 pandemic.

## Method

We conducted a descriptive retrospective cohort study of pregnant women diagnosed with gestational diabetes, Type 1 diabetes mellitus, or Type 2 diabetes mellitus during the COVID-19 pandemic, and assessed their newborns between April 2020 and December 2022 at the King Saud University Medical City, Riyadh, Saudi Arabia. Participants were identified from the electronic medical records. The King Saud University Institutional Review Board approved this study (approval number: E-23-7514). Women who were not tested and infants with congenital anomalies, syndromes leading to hypoglycemia, trisomies, or congenital liver disease were excluded.

We defined gestational diabetes according to the International Association of Diabetes and Pregnancy Study Group (IADPSG) definition, which is the presence of at least one value equal to or above the thresholds of fasting plasma glucose of 5.1 mmol/L, 1-hour plasma glucose level of 10 mmol/L, or 2-hour plasma glucose level of 8.5 mmol/L after a 75-g oral glucose tolerance test (OGTT). We defined a COVID-19-positive mother as one who had a positive COVID-19 swab in the third trimester, or at least 24 h before delivery. All pregnant women were tested for COVID-19 upon admission for delivery, as a hospital policy during the COVID-19 pandemic. 20

The primary outcome was the prevalence of neonatal hypoglycemia in newborns of mothers with diabetes who tested positive for COVID-19. Hypoglycemia was defined as a blood glucose level of less than 2.6 mmol/L in the first 48 h of life, according to the Pediatric Endocrine Society. 21 As part of the hospital policy, all newborns delivered to mothers with diabetes were screened for hypoglycemia at 1 h of life.

The secondary outcome was to assess the short-term effects of hypoglycemia severity in newborns born to diabetic mothers infected with COVID-19, including the need for NICU admission, central-line insertion, length of hospital stay, early- and late-onset sepsis, congenital anomalies, and mortality.

Data were collected from electronic medical records and included maternal COVID-19 swab results at admission, blood glucose levels at delivery, HbA1c levels, type of diabetes, diabetes treatment, COVID-19 treatment, preeclampsia, antenatal steroid use, positive Candida or Guillain–Barré syndrome swabs, rupture of membranes, and length of hospital stay. We then collected data on the newborns, including gestational age, sex, birth weight, mode of delivery, presence of hypoglycemia, NICU admission, need for central lines, congenital anomalies, and NICU length of stay.

### Statistics

Continuous variables are summarized as means (standard deviations), and comparisons between groups were examined using independent *t* -tests, as appropriate. Categorical variables are summarized as frequencies and percentages, and comparisons between groups were examined using chi-square tests (or Fisher’s exact tests, when appropriate). All analyses were conducted using SPSS v29 (IBM Corporation, Armonk, NY), and statistical significance was set at *p* < 0.05.

## Results

Out of 669 mothers with diabetes, 23 were COVID-19 positive at the time of delivery. The mean age was 33.4 years and 33.7 years in the COVID-19-negative and COVID-19-positive patients, respectively. The mean glucose and HbA1c levels were similar between the two groups. Among the mothers, 16 had Type 1 diabetes mellitus, 63 had Type 2 diabetes mellitus, and 590 had GDM, with the majority managed through diet. Fifteen mothers in the COVID-19-negative group tested positive for COVID-19 during the first or second trimesters of pregnancy. Most women who were COVID-19 positive were treated with paracetamol ( Table 1 ).

**Table 1:** Demographic characteristics of mothers included in the study.

Variable	COVID-19 negative	COVID-19 positive	*p* -Value
Number of mothers ( *n* = 669)	646	23	
Age (mean)	33.43	33.7	0.815
Blood sugar at delivery (mean)	5.11	5.63	0.081
HbA1c at delivery (mean)	5.72	5.97	0.202
Rupture of membranes (h) (mean)	2.9	0.52	0.510
Hospital length of stay (h) (mean)	66.44	76.17	0.319
Type of diabetes			0.337
Type 1 DM ( *n* = 16)	15 (2.3%)	1 (4.3%)	
Type 2 DM ( *n* = 59)	59 (9.1%)	4 (17.4%)	
GDM ( *n* = 572)	572 (88.5%)	18 (78.3%)	
Treatment			0.179
No treatment	3 (0.5%)	0	
Insulin	99 (15.3%)	6 (26.1%)	
Oral hypoglycemic agent	77 (11.9%)	5 (21.7%)	
Diet	467 (72.3%)	12 (52.2%)	
Preeclampsia (y)	20 (3.1%)	1 (4.3%)	0.526
Antenatal steroid (y)	63 (9.8%)	21 (91.3%)	>0.994
COVID treatment			<0.001
None	630 (97.5%)	2 (8.7%)	
Paracetamol	13 (2.0%)	21 (91.3%)	

Abbreviations: COVID, coronavirus disease; DM, diabetes mellitus; GDM, gestational diabetes mellitus.

The characteristics of the newborns are shown in Table 2 . The mean gestational age in the COVID-19-positive group was 37.7 weeks, whereas that in the COVID-19-negative group was 38 weeks. The mean birth weight was appropriate for gestational age in both groups. We found that 151 of the 669 women delivered by emergency cesarean section, and 119 delivered by elective cesarean section. However, most women delivered via spontaneous vaginal route.

**Table 2:** Demographic characteristics of newborns.

Variable	COVID-19 negative ( *n* = 646)	COVID-19 positive ( *n* = 23)	*p-* Value
Gestational age (mean)	38	37.7	0.479
Birth weight (kg)	3	3.2	0.218
Mode of delivery			0.421
Emergency CS	143 (22.1%)	8 (34.8%)	
Elective CS	114 (17.6%)	5 (21.7%)	
Assisted delivery	52 (8.0%)	1 (4.3%)	
Vaginal delivery	337 (52.2%)	9 (39.1%)	
Gender (female)	294 (45.5%)	12 (52.2%)	0.528

Abbreviations: COVID-19, coronavirus disease; CS, cesarean section.

Among all the newborns, 128 (18.6%) in the COVID-19-negative group and 8 (34.8%) in the COVID-19-positive group experienced hypoglycemia. Of the 128 newborns with hypoglycemia, 105 required NICU admission for hypoglycemia and other causes. Most newborns received an intravenous dextrose infusion as a treatment for hypoglycemia, with only five requiring glucagon. Central lines were inserted in 16 newborns. Additionally, four newborns were diagnosed with early- or late-onset sepsis. The mean NICU length of stay was 12.9 days for newborns born to COVID-19-negative mothers and 7 days for those born to COVID-19-positive mothers. There was one intrauterine fetal demise in the COVID-19-negative mothers’ group ( Table 3 ).

**Table 3:** Outcome of the newborns.

Variable	COVID-19 negative ( *n* = 646)	COVID-19 positive ( *n* = 23)	*p-* Value
Hypoglycemia (y)	120 (18.6%)	8 (34.8%)	0.061
NICU admission (y)	99 (15.3%)	6 (26.1%)	0.236
Glucose level at admission (mean)	3.3	2.9	0.469
Treatment: IV dextrose	54 (96.4%)	2	>0.994
Treatment: glucagon	5	0	
Central line (y)	15 (15.3%)	1 (16.7%)	>0.994
NICU length of stay (mean/d)	12.9	7	0.442

Abbreviations: COVID-19, coronavirus disease 2019; IV, intravenous; NICU, neonatal intensive care unit.

## Discussion

This is the only cohort study in Saudi Arabia that assessed the short-term outcomes of neonates born to diabetic mothers with positive COVID-19 test results. A large number of pregnant women with diabetes were included over nearly 2 years, and most had GDM. However, we could not establish a relationship between GDM or new-onset diabetes, and COVID-19. This lack of association might be due to the small number of mothers who were COVID-19-positive and the fact that not all COVID-19 positivity equates to active infection.

The higher rate of hypoglycemia observed in the COVID-19-positive group (34.8 vs. 18.6%), although not statistically significant ( *p* = 0.061), warrants discussion of the underlying pathophysiological mechanisms. Several biological pathways may explain how concurrent COVID-19 infection contributes to neonatal hypoglycemia in infants born to diabetic mothers. The SARS-CoV-2 virus gains cellular entry through ACE2 receptors, which are highly expressed on pancreatic islet cells, and direct viral tropism for these cells has been demonstrated, leading to β-cell dysfunction and dysregulated insulin secretion. 14 15 This can exacerbate maternal hyperglycemia, which in turn drives fetal hyperinsulinism through increased transplacental glucose delivery. At birth, the abrupt cessation of the maternal glucose supply leaves the hyperinsulinemic neonate vulnerable to rapid glucose depletion. 16

Furthermore, the cytokine storm associated with COVID-19 infection, characterized by markedly elevated levels of IL-6, TNF-α, and C-reactive protein, induces systemic insulin resistance in the mother, compounding the existing insulin resistance of pregnancy and diabetes. 17 Cortisol-mediated stress hyperglycemia through activation of the HPA axis represents an additional mechanism by which maternal infection may amplify glycemic excursions. 19 Importantly, in our cohort, the majority of COVID-19-positive mothers were treated only with paracetamol (91.3%), suggesting that the observed trend toward higher neonatal hypoglycemia rates is more likely attributable to the direct and indirect metabolic effects of the infection itself rather than iatrogenic causes such as corticosteroid therapy. 18 These mechanisms are not mutually exclusive and may act synergistically in the setting of preexisting maternal diabetes.

We found that 128 newborns developed hypoglycemia within the 1 h of life, most of which was corrected with formula feeding. Only 20 newborns required NICU admission. This may be related to the delay in breastfeeding during the COVID-19 pandemic and the high number of emergency cesarean sections, which may have delayed breastfeeding. Two of the 20 infants required treatment with glucagon in addition to intravenous dextrose. Moeller and colleagues showed that the 1-hour glucose level is the lowest, with glucose levels studied at various intervals from birth to 96 h of age. 22 According to the Canadian Pediatric Society, full-term or late preterm babies at risk of hypoglycemia (e.g., infants born to diabetic mothers) should begin feeding immediately after birth and have their glucose levels screened at 2 h of life, 23 as most term infants stabilize their glucose levels after 3 h of life. However, the glucose level at 24 h of life in term healthy infants is lower than that on the second day of life and after birth. 24

We found no significant differences in the number or causes of admissions (such as respiratory distress syndrome, prematurity, congenital anomalies, sepsis, birth trauma, death, or other factors) between neonates born to diabetic COVID-19-positive mothers and COVID-19-negative mothers. The small number of COVID-19-positive diabetic mothers may have affected these results.

It is noteworthy that the mean HbA1c levels in both groups were below 6% (5.72% in the COVID-19-negative group and 5.97% in the COVID-19-positive group). This finding can be explained by several factors. The vast majority of our cohort (85.5%) had GDM rather than preexisting diabetes, and most were managed by diet alone (72.3% in the COVID-19-negative group). GDM is frequently characterized by isolated postprandial hyperglycemia, which may not substantially elevate HbA1c values. Additionally, physiological changes during pregnancy, including hemodilution and increased red blood cell turnover, are known to lower HbA1c levels by approximately 0.5%, making HbA1c an imperfect marker of glycemic control in pregnant populations 25 . Furthermore, the diagnosis of GDM is based on OGTT thresholds using IADPSG criteria, not on HbA1c, and women can meet diagnostic criteria while maintaining near-normal average glucose levels.

Regarding the delivery mode, we observed a large number of emergency C-sections during the study period. Further investigation is warranted, and these findings should be communicated to the OB-GYN team for comparison with those of previous years. A quality improvement project could explore the reasons behind this trend, as emergency C-sections can negatively affect both mothers and infants, including risks associated with general anesthesia, transient tachypnea of the newborn, hypoglycemia, and bonding issues.

## Limitations

This was a descriptive retrospective cohort study. Although we included a large number of mothers with diabetes, the number of COVID-19-positive cases was small. We included all types of diabetes; while uncontrolled GDM can progress to severe hypoglycemia requiring NICU admission, Type 1 DM carries a higher risk. For hypoglycemia, confounders such as prematurity and hypoxic-ischemic encephalopathy can also contribute to lower glucose levels. Additionally, data on polycythemia, a recognized complication in infants born to diabetic mothers that can itself contribute to hypoglycemia through increased glucose consumption by the elevated red blood cell mass, were not routinely collected and represent a limitation of the current study. Future prospective studies should include hematocrit measurements to evaluate the contribution of polycythemia to neonatal morbidity in this population. 26

## Conclusion

In conclusion, although the study included a large number of mothers with diabetes, especially those with GDM, the number of COVID-19-positive cases was small. Hypoglycemic events were common within the 1 h of life, but most resolved with established feeding. We recommend initiating feeding immediately after birth and screening for hypoglycemia at 2 h of life. No significant differences in short-term neonatal outcomes were observed between diabetic mothers who were COVID-19 positive and those who were COVID-19 negative.

## Data Availability

Datasets are available from the corresponding author on reasonable request.
